# MICAL1 facilitates breast cancer cell proliferation via ROS‐sensitive ERK/cyclin D pathway

**DOI:** 10.1111/jcmm.13588

**Published:** 2018-03-10

**Authors:** Wenjie Deng, Yueyuan Wang, Shuo Zhao, Yujie Zhang, Yan Chen, Xuyang Zhao, Lei Liu, Shixiu Sun, Lin Zhang, Bixing Ye, Jun Du

**Affiliations:** ^1^ Department of Physiology Nanjing Medical University Nanjing China; ^2^ Jiangsu Key Lab of Cancer Biomarkers, Prevention and Treatment Collaborative Innovation Center For Cancer Personalized Medicine Nanjing Medical University Nanjing China; ^3^ Department of Biochemistry and Molecular Biology Nanjing Medical University Nanjing China

**Keywords:** breast cancer, ERK, MICAL1, proliferation, ROS

## Abstract

Molecule interacting with CasL 1 (MICAL1) is a multidomain flavoprotein mono‐oxygenase that strongly involves in cytoskeleton dynamics and cell oxidoreduction metabolism. Recently, results from our laboratory have shown that MICAL1 modulates reactive oxygen species (ROS) production, and the latter then activates phosphatidyl inositol 3‐kinase (PI3K)/protein kinase B (Akt) signalling pathway which regulates breast cancer cell invasion. Herein, we performed this study to assess the involvement of MICAL1 in breast cancer cell proliferation and to explore the potential molecular mechanism. We noticed that depletion of MICAL1 markedly reduced cell proliferation in breast cancer cell line MCF‐7 and T47D. This effect of MICAL1 on proliferation was independent of wnt/β‐catenin and NF‐κB pathways. Interestingly, depletion of MICAL1 significantly inhibited ROS production, decreased p‐ERK expression and unfavourable for proliferative phenotype of breast cancer cells. Likewise, MICAL1 overexpression increased p‐ERK level as well as p‐ERK nucleus translocation. Moreover, we investigated the effect of MICAL1 on cell cycle‐related proteins. MICAL1 positively regulated CDK4 and cyclin D expression, but not CDK2, CDK6, cyclin A and cyclin E. In addition, more expression of CDK4 and cyclin D by MICAL1 overexpression was blocked by PI3K/Akt inhibitor LY294002. LY294002 treatment also attenuated the increase in the p‐ERK level in MICAL1‐overexpressed breast cancer cells. Together, our results suggest that MICAL1 exhibits its effect on proliferation via maintaining cyclin D expression through ROS‐sensitive PI3K/Akt/ERK signalling in breast cancer cells.

## 1 INTRODUCTION

Molecules interacting with casL (MICALs) are multidomain redox enzymes that are able to sever F‐actin filaments and decrease its polymerization via direct oxidation of actin.^1^, ^2^, ^3^ They are widely expressed in nervous system and other tissues, including endothelial cells and cancer cells such as melanoma and HeLa cells.^4^, ^5^, ^6^, ^7^ Although MICAL family is identified as MICAL (1‐3) and MICAL‐like (‐L1, ‐L2) forms in mammals, its main functions were studied mostly in Drosophila.^1^, ^3^, ^8^ Normally, MICAL family members have four conserved domains: N‐terminal flavin adenine dinucleotide (FAD) binding domain, Lin11, Isl‐1 and Mec‐3 (LIM) domain, calponin homology (CH) domain and C‐terminal coiled‐coil (CC) domain. FAD domain contains flavin mono‐oxygenase activity and is responsible for majority of MICAL1's function.^9^ Recently, overexpression of MICAL2 and MICAL‐L2, the other members of MICAL family, has been confirmed to be related to multiple invasive phenotype of cancer cells such as increased motility, proliferation, as well as inducing epithelial‐to‐mesenchymal transition (EMT).^10^, ^11^ Domain architecture of MICAL1 is closely related to Drosophila MICAL^4^; however, to date, only a few reports characterizing the functions of MICAL1 in human cancer progression have been published.

Sustaining proliferative signalling and resistant cell death are important hallmarks of cancer.^12^ More and more cellular molecules are identified as essentials for regulating those progresses.^13^, ^14^, ^15^ Previous studies have reported the anti‐apoptosis effect of MICAL1 in human melanoma cells. The mechanism was demonstrated to be associated with MICAL1's negative control of mammalian Ste‐20‐like kinase 1 (MST1)‐nuclear‐Dbf2‐related kinase (NDR) apoptotic signalling by competing with MST1 for NDR binding.^5^, ^16^ Despite its characteristic on anti‐apoptosis, whether MICAL1 could influence cancer cell proliferation and the underlying molecular mechanism remains unclear. Recent immunohistochemical studies revealed that MICAL1 is highly expressed in hBRAFV600E human melanomas which display constitutive activation of the AKT, ERK pathway and abnormal melanoma growth.^5^ MICAL1 has been identified exert its effect on promoting breast cancer cell invasion with RAB protein.^17^ In this study, we will address the role of MICAL1 in breast cancer cell proliferation and provide evidence for a mechanism describing its regulation.

Our previous work provided evidence that MICAL1 plays an essential role in the activation of ROS/Akt signalling and cell invasive phenotype and identified a novel link between RAB35 and MICAL1 in promoting breast cancer cell invasion.^17^ In the current study, our results suggest that MICAL1 exhibits its positively regulatory function on breast cancer cell proliferation via maintaining cyclin D expression through ROS‐sensitive PI3K/Akt/ERK signalling, which implicates an essential role for MICAL1 in breast cancer pathogenesis.

## 2 MATERIALS AND METHODS

### 2.1 Cell and plasmids

Human breast cancer cell lines MCF‐7 and T47D were originally obtained from the Cell Biology Institute of Chinese Academy of Sciences (Shanghai, China). Cells were cultured in Dulbecco's modified Eagle's medium (DMEM, high glucose) (Hyclone) supplemented with 10% (v/v) foetal bovine serum (FBS) (Hyclone) and antibiotics (100 U/mL streptomycin and 100 μg/mL penicillin) (Invitrogen) in a humidified incubator at 37°C with 5% CO_2_. Cells were grown on coverslips for fluorescence staining and on plastic dishes for protein extraction.

Human MICAL1 cDNA clone was purchased from Youbio (Hunan, China). The full‐length MICAL1 DNA was amplified from pOTB7‐MICAL1 plasmid using the following primer set, sense: 5′‐CCCAAGCTTGCCACCATGGCTTCACCTACCTCCA‐3′, antisense: 5′‐CCAACTCGAGGCCCTGGGCCCCTGTCCCCAAGGCCA‐3′. In these primers, Hind III and XhoI restriction site sequences have been underlined. The PCR products were cloned into the pCMV‐C‐HA vector (Beyotime, Nantong, China). The cells were seeded in 6‐well plates, cultured to 80% ~ 90% confluence and then transfected with plasmids using FuGENE HD transfection reagent (Promega) according to the manufacturer's instructions.

### 2.2 siRNA knockdown studies

The sequences of small interfering RNA (siRNA) for MICAL1 were as follows: #1, 5′‐GUCUCUGCCUUUGACUUCATT‐3′, #2, 5′‐CUGCAGAACAUUGUGUACUTT‐3′ and #3, 5′‐CUCGGUGCUAAGAAGUUCUTT‐3′; the sequence of control siRNA was 5′‐UUCUCCGAACGUGUCACGUTT‐3′ (GenePharma). Cells were transfected with siRNA by Lipofectamine 2000 (Invitrogen) according to the manufacturer's instruction.

### 2.3 MTT assays

Cell viability was determined by 3‐(4,5‐dimethylthiazol‐2‐yl)‐2,5‐diphenyltetrazolium bromide (MTT) assay, as described previously.^18^ In brief, cells were seeded at a density of 5 × 10^3^ cells per well into 96‐well plate and transfected with siRNA or plasmids as indicated. Ten replicas were made for each group. After cultured for the indicated time, cells were washed and MTT (Sigma) was added. The plate was then incubated in the dark for 4 hours, followed by measurement at 490 nm using a microplate absorbance reader (Bio‐Tek, Elx800, USA).

### 2.4 Colony formation assays

In brief, 1 × 10^3^ stably transfected cells were seeded into 6‐well dish and cultured for up to 2 weeks. Colonies were visualized by crystal violet staining, and then, colonies were photographed and counted.

### 2.5 Flow cytometry analysis

Cell cycle analysis was performed by flow cytometry. Briefly, cells were harvested and fixed in 80% ice‐cold ethanol overnight. Then the cells were incubated with RNase A and propidium iodide staining solution at 37°C for 30 minutes in darkness. Subsequently, the stained cells were analysed using flow cytometry.

### 2.6 Cytoplasmic and nuclear protein extraction

Cytoplasmic and nuclear proteins were obtained using the Nuclear and Cytoplasmic Protein Extraction Kit (Beyotime) according to the manufacturer's instructions.^19^ In brief, cells were harvested by centrifugation and resuspended in cytoplasmic extraction agent A. The solution was vigorously vortexed and then incubated on ice for 10 minutes. Then, cytoplasmic extraction agent B was added to the cell pellet. The pellet was vortexed and incubated on ice for 1 minute. The pellet was vortexed again and centrifuged for 5 minutes at 5000 rpm. Supernatant was collected as cytoplasmic extract. The insoluble (pellet) fraction was suspended by nuclear extraction agent. After vortexed several times, the mixture was centrifuged for 10 minutes at 12000 rpm. The supernatant was collected as nuclear extract.

### 2.7 Western blotting assays

Subconfluent cells were washed with PBS and lysed for 20 minutes on ice in a RIPA buffer containing 1% protease inhibitor cocktail (Beyotime). The protein was collected, and its concentration was determined by the BCA protein assay reagent kit (Thermo Fisher Scientific) then separated by SDS‐PAGE and transferred to nitrocellulose membrane. The membrane was then blocked with 5% non‐fat milk for 1 hour at room temperature and incubated with primary antibody overnight at 4°C. The following antibodies were used: GAPDH (Bioworld), MICAL1 (Proteintech) (Santa Cruz), NF‐kB (Santa Cruz), β‐catenin, p‐β‐catenin, p‐GSK‐3β, p‐S6K, histone‐H3, ERK, p‐ERK, Akt and p‐Akt (Cell Signaling), cyclin D (ABclonal), c‐myc, CDK (2, 4, 6) and cyclin (A, E) (Santa Cruz). The membranes were incubated with secondary HRP‐conjugated antibodies (Santa Cruz) and visualized with ECL reagent (Millipore). Digital images of immunoblots were obtained with a Chemidoc XRS and analysed using the image analysis program Quantity One (Bio‐Rad).

### 2.8 Immunofluorescence assays

Cells for immunostaining were fixed in ice‐cold methanol for 20 minutes, then permeabilized in 0.1% Triton X‐100 and blocked in PBS containing 1% BSA (Sigma) for 1 hour at room temperature. The cells were incubated with primary antibody overnight at 4°C followed by incubation with rhodamine‐conjugated secondary antibody (Life Technologies) for 1 hour at room temperature within a moist chamber. After washing with PBS, the samples were mounted with DAPI Fluoromount G (Southern Biotech). Images were acquired using an Olympus BX51 microscope coupled with an Olympus DP70 digital camera, and fluorescence intensity was quantified by ImageJ. Five images in each group were analysed.

### 2.9 5‐Ethynyl‐2‐deoxyuridine (EdU) incorporation assays

Cell proliferation was measured using EdU staining kits (Keygen) according to the manufacturer's instruction. Five replicas were made for each group. In brief, cells were cultured on coverslips until reaching 70% confluence, then EdU was added to the culture media for 2 hours. After labelling, cells were washed three times with PBS followed by formaldehyde fixation. Then, the cells were incubated with glycine and washed with PBS containing 0.5% Triton X‐100. After the cells were counterstained with DAPI, cells were mounted and imaged by fluorescence microscopy (Olympus BX 51, Tokyo, Japan) coupled with an Olympus DP70 digital camera.

### 2.10 Hoechst staining

Cell apoptosis was measured using Hoechst staining according to the manufacturer's instruction (Beyotime). Three replicas were made for each group. In brief, the Hoechst 33258 staining solution was prepared by diluting the Hoechst stock solution 1:2000 in PBS. After removing the medium, sufficient staining solution was added to the cells, and then, the cells were protected from the light for 30 minutes at room temperature. The staining solution was removed and cells were washed with PBS for three times. Observation and photography were performed by a fluorescence microscope.

### 2.11 Measurement of ROS

For intracellular H_2_O_2_ staining, 1 × 10^5^ cells were seeded on a coverslip placed in a 6‐well plate and incubated overnight. After being treated with appropriate inhibitors as detailed elsewhere in the text, the cells were stained with 5 μmol/L 2′,7′‐dichlorofluorescein diacetate (CM‐H_2_DCFDA) (Life Technologies) for 15 minutes at 37°C, washed with PBS three times and fixed with 4% formaldehyde. After washing with PBS, the cover slips were mounted on glass slides. Images were collected using a fluorescence microscope.

### 2.12 Statistical analysis

Statistical analysis was performed using the SPSS. Error bars represent the standard error of mean SEM, and the significance of difference between the two groups was analysed by Student's *t* test. *P *<* *.05 represents statistical significance, and *P *<* *.01 represents sufficiently statistical significance (two‐tailed). All experiments were repeated at least three times independently.

## 3 RESULTS

### 3.1 MICAL1 promotes breast cancer cell proliferation in vitro

**Figure 1:**
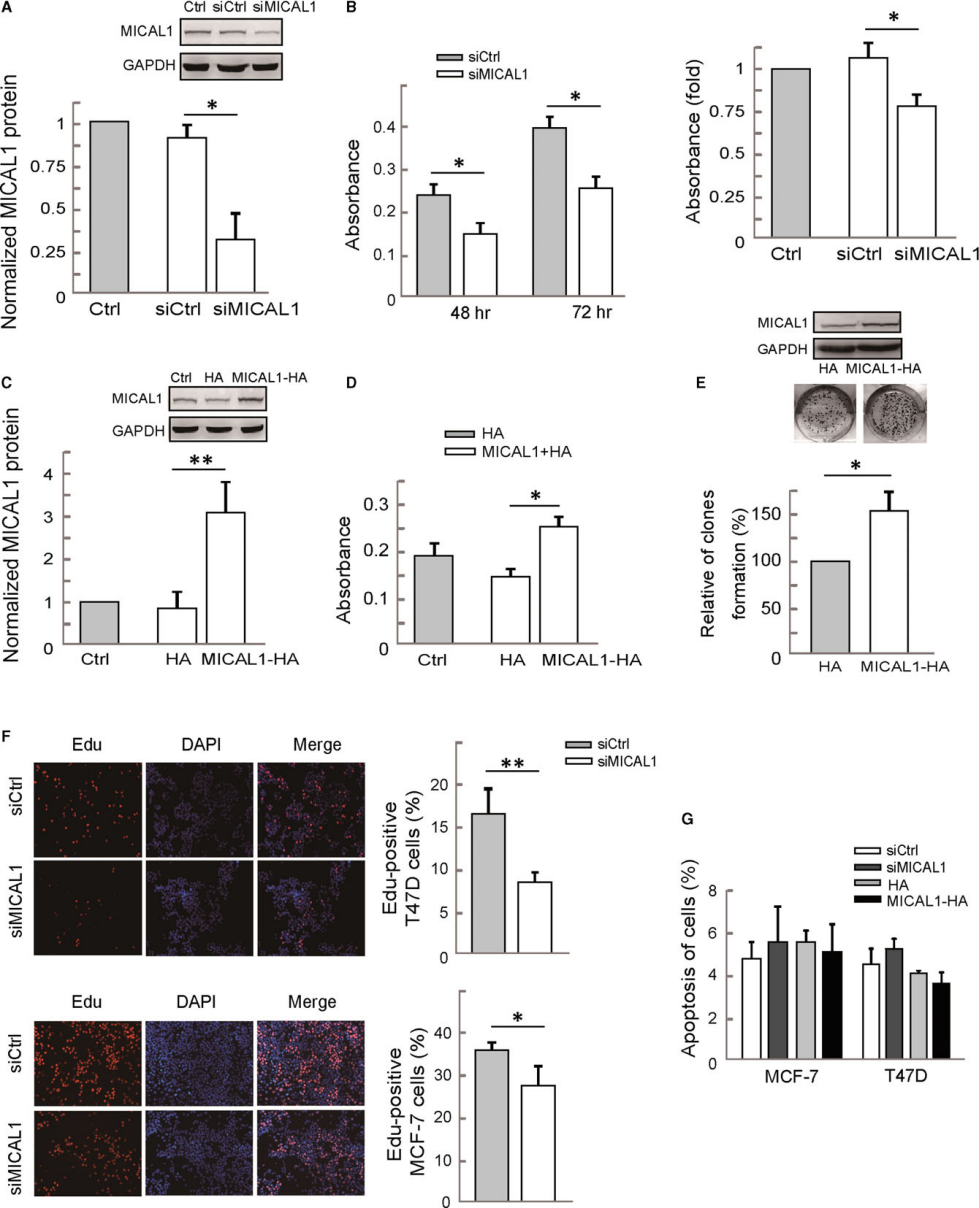
MICAL1 promotes breast cancer cell proliferation. A, MCF‐7 cells were transfected with negative control siRNA or siRNA specifically targeting MICAL1 (siMICAL1). Total protein extracts from cells were analysed by Western blotting analysis for MICAL1 expression. B, MTT assays in MICAL1 knockdown MCF‐7 cells (left) and T47D cells (right). C, T47D cells were transfected with MICAL1 or empty vector and analysed for MICAL1 expression by Western blotting analysis. D, MTT assays in MICAL1‐overexpression T47D cells. E, Stably transfected cells were subjected to colony formation assays and incubated for 14 d. Colonies were photographed and counted. F, Edu staining shows the effect of MICAL1 depletion on cell proliferation of MCF‐7 and T47D. G, Hoechst staining shows the effect of MICAL1 depletion on cell apoptosis. *P <* *.05, **P <* *.01.

To investigate the impact of MICAL1 on cell growth, we first determined the cell growth using MTT assay. The knockdown efficiency of MICAL1 in MCF‐7 cells was determined by Western blotting assays (Figure 1A). We performed the same transfection in MCF‐7 and T47D cells and found that by silencing MICAL1, cell growth rate in both cell lines was inhibited (Figure 1B). In contrast, induced MICAL1 expression resulted in an accelerated cell proliferation rate compared with the controls (Figure 1C,D). The colony formation assay showed that MCF‐7 cells with stable MICAL1 expression increased colony number by 1.5‐fold compared to control cells (*P *<* *.05) (Figure 1E). Edu staining analysis showed that MCF‐7 and T47D cells with low MICAL1 expression exhibited significant reduction in cell proliferation rate (Figure 1F). Hoechst staining showed that MICAL1 did not significantly alter cell apoptosis (Figure 1G). All these results indicated a stimulatory function of MICAL1 in proliferation of breast cancer cells.

### 3.2 MICAL1 depletion decreases expression of cyclin D

**Figure 2:**
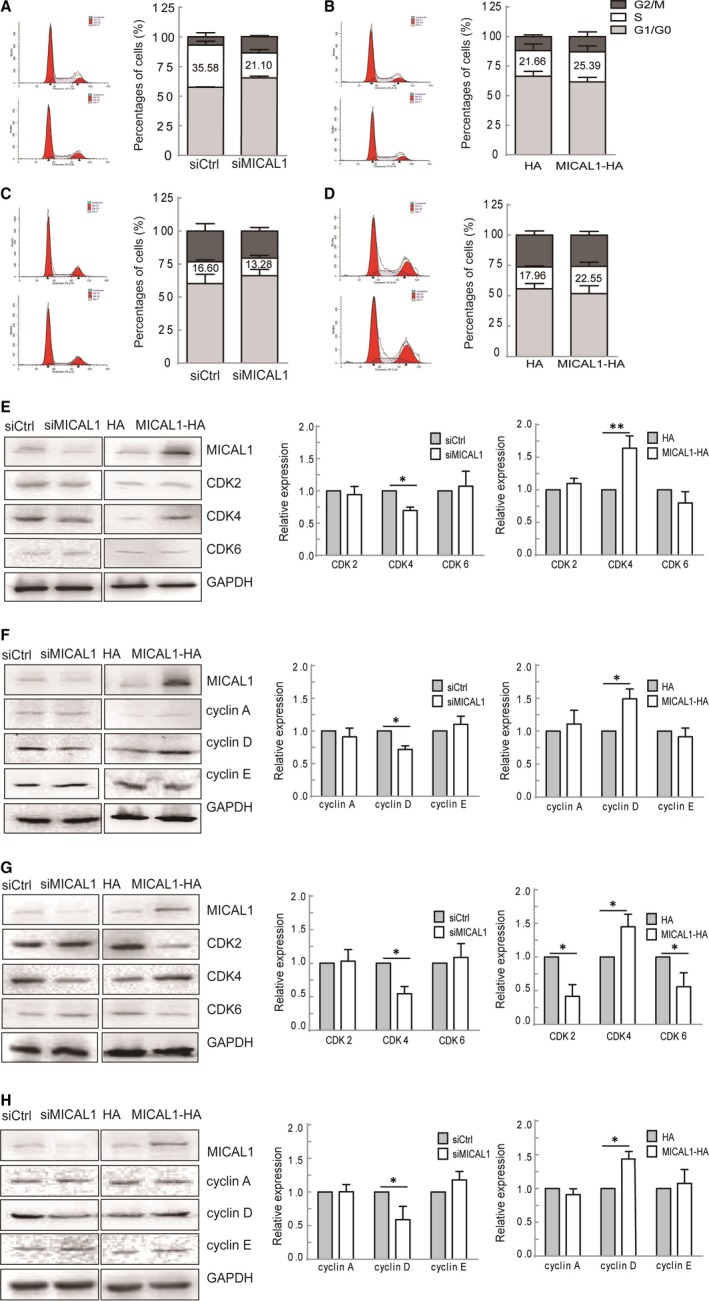
MICAL1‐induced modulations of cell cycle‐related proteins. (A&B) MICAL1 depleted (A) or MICAL1 overexpressed (B) MCF‐7 cells underwent cell cycle analysis by flow cytometry. (C&D) MICAL1 depleted (C) or MICAL1 overexpressed (D) T47D cells underwent cell cycle analysis by flow cytometry. Cell cycle data are shown in histograms. (E&F) MICAL1 depleted (E) or MICAL1 overexpressed (F) T47D cells underwent Western blotting analysis for CDK2, CDK4, CDK6, cyclin A, cyclin D, cyclin E. (G&H) MICAL1 depleted (G) or MICAL1 overexpressed (H) MCF‐7 cells underwent Western blotting analysis for CDK2, CDK4, CDK6, cyclin A, cyclin D, cyclin E. *P <* *.05, **P <* *.01.

To further verify the impact of MICAL1 expression on breast cancer proliferation, we assessed cell cycle distribution. As shown in Figure 2A,C, MICAL1 silencing MCF‐7 or T47D cells demonstrated a slight increase in percentage of cells in the G1 phase and a decrease in percentage of cells in the S phase. Conversely, an increase in the percentage of cells in the S phase and a decrease in the G1 phase were observed in MICAL1 overexpressed MCF‐7 (Figure 2B) or T47D cells (Figure 2D) compared to the control group.

The progression of cell proliferation needs to pass through cell cycle which is strictly regulated by key regulatory proteins. A number of key markers, such as cyclin‐dependent kinases (CDK2, CDK4, CDK6) and cyclin (A, D, E), reportedly dynamically modulate cell cycle. We noticed that, among these molecules, the levels of cyclin D and CDK4 were markedly blocked after silencing MICAL1. Consistently, cyclin D and CDK4 were higher when MICAL1 overexpressed in T47D cells (Figure 2E,F) and MCF‐7 cells (Figure 2G,H). These results indicate that cyclin D and CDK4 play key roles in MICAL1‐induced cell proliferation.

### 3.3 MICAL1‐mediated cell proliferation is independent of wnt/β‐catenin, NF‐κB and mTOR pathways

**Figure 3:**
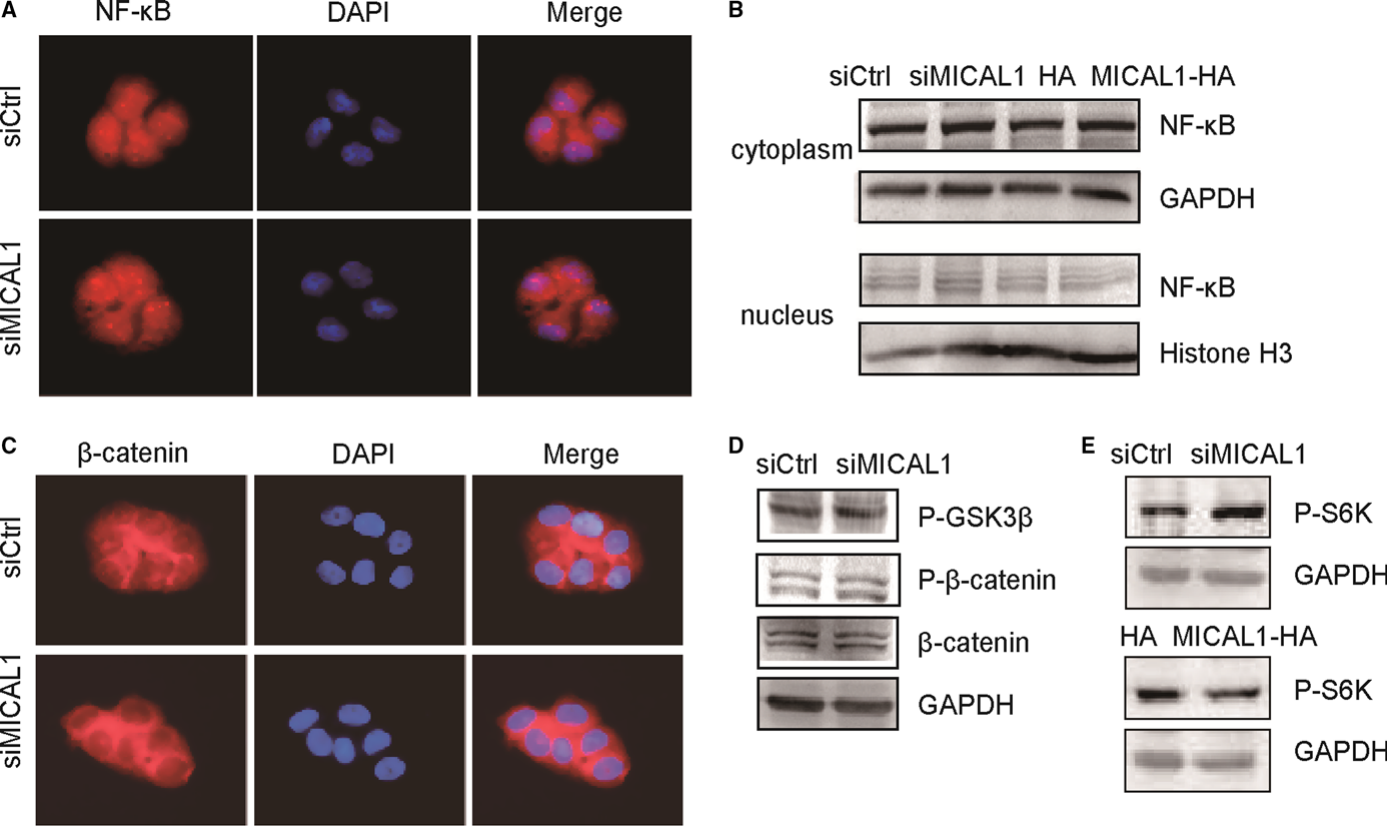
Effect of MICAL1 on wnt/β‐catenin, NF‐κB and mTOR/S6K pathways (A&B) Effects of MICAL1 on NF‐κB distribution in T47D cells were examined by immunofluorescence (A) and Western blotting assays (B). (C&D) Effects of MICAL1 on wnt/β‐catenin signalling in T47D cells were examined by immunofluorescence (C) and Western blotting assays (D). (E) Protein level of p‐S6K in T47D cells were examined after the cells were transfected with siMICAl1 or MICAL1

To determine whether the effects of MICAL1 on cell proliferation were associated with wnt/β‐catenin or NF‐κB pathways, we explored the expressions of key markers in these pathways which were demonstrated to be closely associated with cyclin D expression. Compared to the control group, no significant changes in the expression as well as subcellular location of NF‐κB and β‐catenin were found in MICAL1‐depletion cells (Figure 3A‐C). Furthermore, neither p‐GSK3β nor P‐β‐catenin expression markedly altered after MICAL1 knockdown (Figure 3C,D). These results suggest that MICAL1‐mediated regulation of cell proliferation was not dependent on wnt/β‐catenin and NF‐κB pathways. In addition, p‐S6K, the mTOR pathway effector related to cell proliferation decreased after MICAL1 overexpression and increased after MICAL1 depletion (Figure 3E), suggesting that the MICAL1 function in breast cancer cell proliferation was probably not mediated by mTOR/S6K signalling.

### 3.4 Involvement of p‐ERK signalling in MICAL1‐induced proliferation

**Figure 4:**
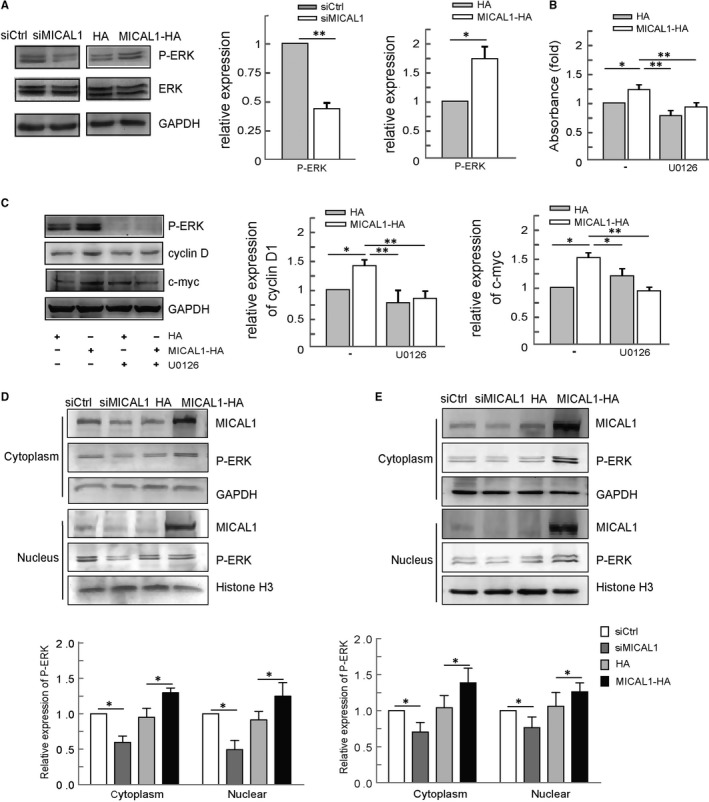
Effect of MICAL1 on ERK/cyclin D pathways. (A) Effect of MICAL1 on ERK activation. (B) T47D cells transfected with MICAL1 were treated with 1 μmol/L U0126 for 24 h, and cell survival was examined by MTT assay. (C) T47D cells transfected with MICAL1 were treated with 1 μmol/L U0126 for 24 h, and protein levels of c‐myc and cyclin D were examined. (D&E) Effect of MICAL1 on p‐ERK distribution in T47D (D) and MCF‐7 cell (E) was examined by Western blotting assays. *P <* *.05, **P <* *.01.

Next, we examined the effect of p‐ERK inhibitor U0126 on cyclin D expression and cell proliferation. As shown in Figure 4A, overexpression of MICAL1 resulted in increased cyclin D in breast cancer cells. In addition, the down‐regulated expression of cyclin D was induced by silencing of MICAL1. Here, increased cell proliferation rate (Figure 4B) as well as higher expression level of cyclin D and c‐myc was found in MICAL1 overexpression cells, and this change was markedly blocked after treatment with U0126 (Figure 4C). As shown in Figure 4D,E, lower levels of p‐ERK were found both in cytoplasm and in nucleus parts in the MICAL1‐depletion T47D and MCF‐7 cells. In contrast, the expression of p‐ERK in the MICAL1‐overexpression T47D and MCF‐7 cells was increased greatly when compared to the control group. Combined with the above results, it suggests that MICAL1 may support ERK activation and then increases cyclin D expression which is involved in cell cycle and proliferation regulation in breast cancer cells.

### 3.5 ROS mediates ERK/cyclin D signals via phosphorylated Akt

**Figure 5:**
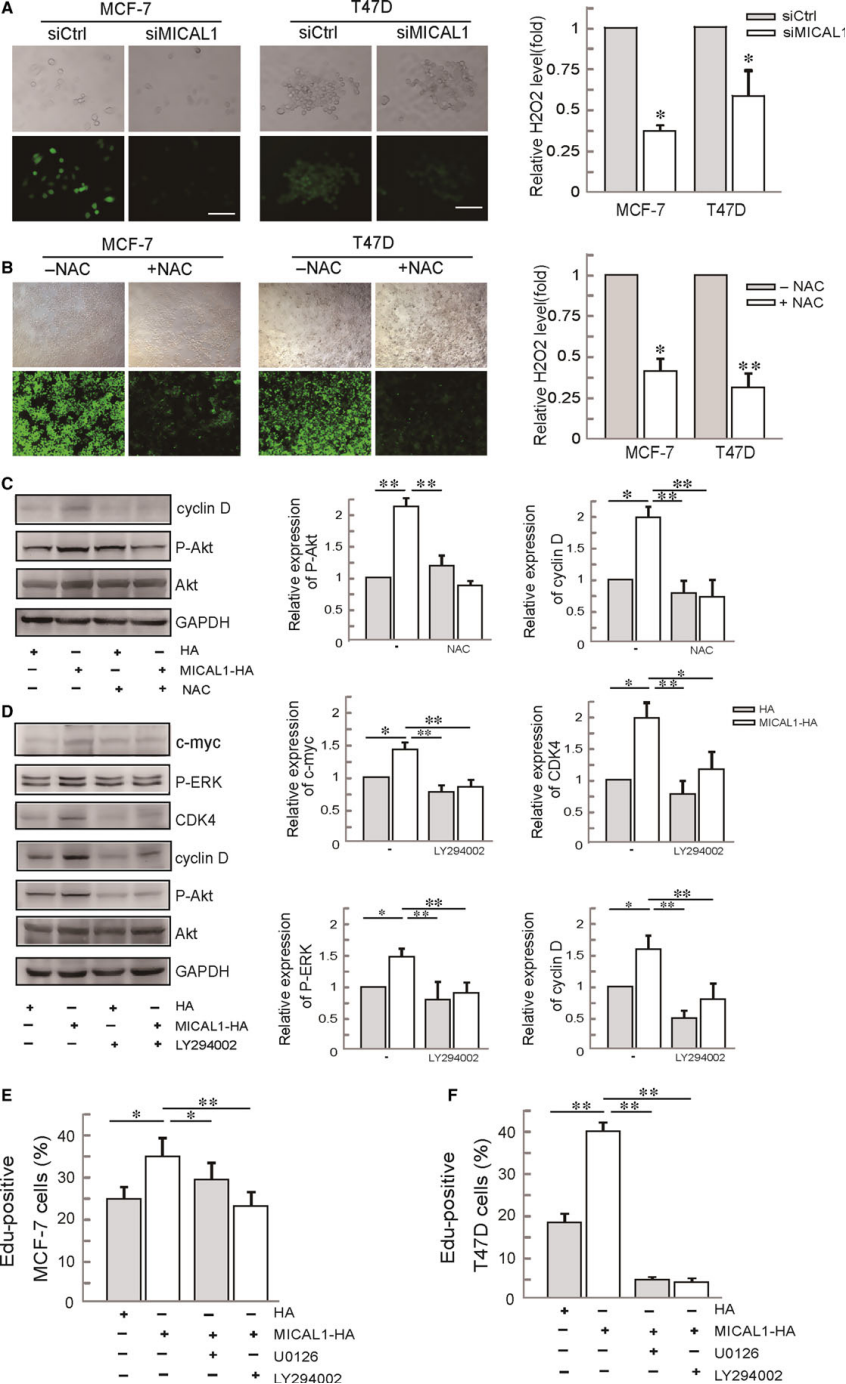
Effects of ROS on MICAL1‐modulated Akt activity and ERK/cyclin D signalling. (A) Effects of MICAL1 on ROS generation in MCF‐7 and T47D cells. *P <* *.05 the cells transfected with siMICAL1 relative to the cells with control siRNA. (B) Effects of NAC on ROS generation in MCF‐7 and T47D cells. *P <* *.05, **P <* *.01 the NAC‐treated group with relative to the control group. (C) T47D cells transfected with MICAL1 and treated with 2 mmol/L NAC for 1 h, protein levels of p‐Akt, cyclin D were examined. (D) T47D cells transfected with MICAL1 were treated with 1 μmol/L LY294002 for 30 min, and protein levels of p‐ERK, c‐myc, CDK4, cyclin D were examined. (E&F) Edu staining shows the effects of U0126 and LY294002 on proliferation in MICAL1‐transfected MCF‐7 (E) and T47D (F) cells. *P <* *.05. **P <* *.01

**Figure 6:**
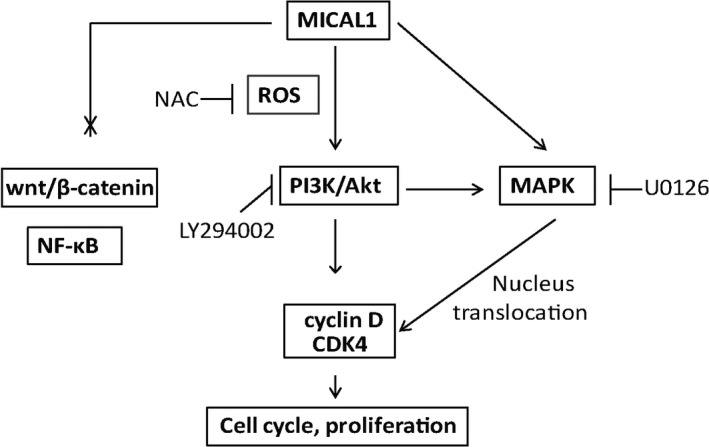
A model for the role of MICAL1 in breast cancer cell proliferation. MICAL1 promotes proliferation by promoting CDK4 and cyclin D expressions via ROS‐sensitive PI3K/Akt/ERK signalling

MICAL1 is well characterized in ROS generation, which has been associated with cancer cell invasion.^17^ We transfected MCF‐7 and T47D cells with siMICAL1 and then examined its effects on ROS production. As shown in Figure 5A, ROS level in those cells was inhibited by siMICAL1 transfection. ROS scavenger NAC was used here to eliminate ROS production (Figure 5B). To probe the involvement of PI3K/Akt activation in MICAL1‐induced cell proliferation, we transfected T47D cells with MICAL1 and p‐Akt expression was detected by Western blotting assays. Our observations yielded evidence that the level of p‐Akt was markedly increased after the overexpression of MICAL1. Moreover, pre‐treatment with NAC inhibited p‐Akt level when MICAL1 was overexpressed (Figure 5C). NAC and LY294002 pre‐treatment also delayed the increased p‐ERK, c‐myc and cyclin D levels induced by MICAL1‐overexpression (Figure 5C,D). MICAL1‐induced cell proliferation was also inhibited by U0126 and LY294002 in both MCF‐7 and T47D cells (Figure 5E,F). Collectively, these data indicated that MICAL1 may facilitate ROS generation, which leads to PI3K/Akt/ERK/cyclin D signalling activation and breast cancer cell proliferation (Figure 6).

## 4 DISCUSSION

MICALs are multidomain proteins previously known for their roles in organization of cytoskeleton and synaptic structures. For example, Drosophila MICAL is identified as an essential for neuronal growth by interactions with the cytoplasmic region of plexin and activating plexin‐ and semaphorin‐mediated axonal signalling.^20^ Recent studies also reported that MICAL1 promoted the development of hippocampal mossy fibre connections through its ability to control actin cytoskeleton rearrangement.^21^ The notion that cell cytoskeleton rearrangement contributes to cell proliferation has been confirmed by many research groups^15^; however, up to now, no study yet addressed the function of MICAL1 in the human cancer cell proliferation process. In contrast to previous findings which indicated that the expression of MICAL‐L2, another member of MICAL family, is related to the clinical stage and histologic grade of ovarian cancer,^11^ this study showed a novel link between MICAL1 and cell proliferation for the first time. Therefore, it is interesting to investigate the signalling mechanisms underlying the effect of MICAL1 on promoting breast cancer cell proliferation.

Cyclin‐dependent kinase is protein kinase that could regulate cell cycle by binding to cyclin. CDK4‐Cyclin D complex is a major integrator of cell proliferation which is required for progression through G1 phase and entry into S phase of the cell cycle. Here, we noticed that knockdown of MICAL1 in breast cancer cells significantly down‐regulated both CDK4 and cyclin D protein levels, whereas MICAL1 overexpression could reverse those effects on CDK4 and cyclin D. In addition, the numbers of breast cancer cell in S phase of the cell cycle were significantly lower after silencing of MICAL1 compared with control group, while MICAL1 overexpression improved them. The above results indicate that MICAL1 expression may lead to an increase in CDK4‐Cyclin D complex that stimulates cellular G1/S transition, which is probably involved in MICAL1‐induced breast cancer cell proliferation.

Cyclin D is regulated by the upstream pathways including NF‐κB, PI3K/Akt/mTOR and Wnt/β‐catenin.^22^, ^23^, ^24^, ^25^, ^26^ MICAL1 has an important role in triggering Akt phosphorylation, which is a key signal that can endow breast cancer cells with an invasive phenotype.^17^ We have noticed that MICAL1 selectively activates PI3K/Akt signalling pathway. To our surprise, p‐S6K protein level, the main downstream effector of PI3K/Akt/mTOR cascade, was increased after MICAL1 depletion and decreased after MICAL1 overexpression, suggesting that mTOR may not the target of MICAL1 for proliferation regulation. To find out whether the effects of MICAL1 were dependent on Wnt/β‐catenin and NF‐κB signalling pathways or not, we explored the expression of p‐β‐catenin, p‐GSK‐3β and the distribution of NF‐κB in cytoplasm and nucleus. Results showed that MICAL1 depletion did not significantly alter expression or location of those markers, indicating that the inhibitory effects of MICAL1 were also independent on those signalling pathways. MAPKs are highly conserved kinases that are critical for communicate signals from cell surface to DNA in the nucleus. Among the members of the MAPK family, extracellular signal‐regulated protein kinase (ERK) can alter the level and activity of transcription factor c‐myc, leading to changes in cyclin D transcription that are mainly responsible for cell cycle.^27^ Phosphorylation of ERK and its translocation to nucleus by ROS have been proven to be an important mechanism to mediate breast cancer cell migration by LPA.^28^ We detected an up‐regulation of p‐ERK in the nucleus upon MICAL1 expression. In addition, inhibition of ERK activation by U0126 results in an decrease in cyclin D expression in MICAL1‐overexpressed cells, indicating that MICAL1‐induced breast cancer cell proliferation might be due to cyclin D expression by a p‐ERK dependent way.

More and more evidence showed that MICAL uses its FAD domain to either oxidize proteins or produce ROS such as H_2_O_2_. MICAL proteins may oxidize actin at methionine residues directly, causing depolymerization of actin filaments.^8^ Microtubule assembly can also be altered indirectly by the ROS produced by MICAL.^29^ Oxidative stress originates from an imbalance between the generation and scavenging of ROS, activates aberrant signalling cascades and leads to tumorigenesis. A recent study reported that increased ROS production was triggered by overexpression of constitutively active MICAL1 mutants.^16^ On the contrary, ROS levels were significantly attenuated upon transfection of the enzymatically impaired FAD domain mutant.^4^ Consistent with these findings, we found that knockdown of MICAL1 decreased the production of ROS, suggesting that ROS may be a major downstream effect of MICAL1. PI3K/Akt and ERK are particularly sensitive to redox reaction.^28^, ^30^ Additionally, ROS scavenger NAC significantly reversed MICAL1 overexpression induced up‐regulation of p‐Akt and p‐Akt inhibitor LY294002 significantly reversed MICAL1 overexpression induced up‐regulation of p‐ERK, CDK4 as well as cyclin D in breast cancer cells. In summary, these data suggest that MICAL1 may promote breast cancer cell proliferation by ROS‐PI3K/Akt signalling pathway activation.

The present study was a continuation of our previous study where we have proven that ROS‐PI3K/Akt signalling pathway is selectively responsible for MICAL1‐induced breast cancer cell invasion,^17^ providing a basis for further exploring the role of PI3K/Akt signalling in MICAL1‐induced malignant phenotype. In this study, we examined the effects of MICAL1 on breast cancer cell proliferation and found that MICAL1 exhibits its effects by regulating cyclin D expressions via ROS‐sensitive PI3K/Akt/ERK signalling. Our observations described MICAL1's effect on breast cancer cell proliferation and may help to better understand how deregulation of MICAL1 contributes to breast cancer progression.

## CONFLICT OF INTEREST

The authors confirm that there are no conflicts of interest**.**

## AUTHORS’ CONTRIBUTIONS

JD designed the study. WD, YW, YZ, YC, LL and SS performed the experiments. YW, XZ, SZ, LZ, BY performed the statistical analysis. JD drafted the manuscript. All authors read and approved the final manuscript.
